# Plasma Electrolytic Oxidation for Dental Implant Surface Treatment

**DOI:** 10.17691/stm2023.15.3.02

**Published:** 2023-05-28

**Authors:** A.A. Muraev, A.I. Murzabekov, S.Yu. Ivanov, Yu.V. Tarasov, E.A. Orlov, A.A. Dolgalev

**Affiliations:** Professor, Department of Oral and Maxillofacial Surgery; Peoples’ Friendship University of Russia (RUDN University), 6 Miklukho-Maklaya St., Moscow, 117198, Russia;; Oral Surgeon, Head of the Center for Medical Care in Dental Diseases; The Central Clinical Hospital with a Polyclinic of the Presidential Administration of the Russian Federation, 15 Marshal Timoshenko St., Moscow, 121359, Russia;; Professor, Correspondent Member of Russian Academy of Sciences, Head of the Department of Oral and Maxillofacial Surgery; Peoples’ Friendship University of Russia (RUDN University), 6 Miklukho-Maklaya St., Moscow, 117198, Russia; Head of the Department of Maxillofacial Surgery; I.M. Sechenov First Moscow State Medical University, 8/2 Trubetskaya St., Moscow, 119991, Russia;; General Director; Beta-Tech Medicine LLC, Bldg 1, 42 Bolshoy Boulevard, Mozhaisky District, Skolkovo Innovation Center, Moscow, 121205, Russia;; Head of the Laboratory; Beta-Tech Medicine LLC, Bldg 1, 42 Bolshoy Boulevard, Mozhaisky District, Skolkovo Innovation Center, Moscow, 121205, Russia;; Professor, Department of General Dental Practice; Stavropol State Medical University, 310 Mira St., Stavropol, 355017, Russia

**Keywords:** dental implant, osteointegration, implant surface treatment techniques, plasma electrolytic oxidation, hydroxyapatite, calcium hydroxyphosphate

## Abstract

Current technologies of plasma electrolytic oxidation (PEO) for modifying the surfaces of dental implants made of the Grade IV titan alloy provide predictable long-term results in implant dentistry.

**The aim of the study** is to evaluate the efficacy of PEO technology comparing two types of surface modification of dental implants made of VT1-0 medical titanium alloy.

**Materials and Methods:**

50 IRIS dental implants (Scientific Production Company LICOSTOM, Russia), 10-mm long and 4 mm in diameter, were manufactured from the VT1-0 alloy. The implant surface was treated by two PEO methods: 1) in the aqueous solution of alkaline electrolyte without any additional modifiers (PEO-Ti); 2) in the aqueous solution of orthophosphoric acid-based electrolyte containing calcium carbonate (PEO-Ca). Implants made of VT1-0 alloy after milling and without additional treatment served as control samples. The implant surfaces were studied by electron microscopy and energy dispersive X-ray spectrometry. Some of the implants were installed in sheep, samples were obtained at 2, 4, and 8 weeks and studied by microcomputer tomography.

**Results:**

Regardless of the electrolyte composition, a highly developed porous surface was formed in the samples with PEO-modified surfaces. The surface of the PEO-Ti samples in a simple unmodified electrolyte was characterized by a large number of open pores with a wide range of size distribution from 200 nm to 3 μm. The pore size distribution was of a monomodal character, with a maximum near 0.23 μm. The PEO samples in the Ca-containing electrolyte had pores also in a wide range from ~80 nm to ~7 μm. The pore distribution, in contrast to PEO-Ti, was bimodal in nature, with the main maximum in the region of 1.05 μm and the concomitant maximum near 2.45 μm.

The obtained surfaces of both types (PEO with Ca and Ti) possessed high purity and optimal microroughness for osseointegration. Both types of PEO treatment (PEO with Ca and Ti) have demonstrated a similar osseointegrative potential, nevertheless, the surface of the PEO-Ca showed a better contact with the implant surface (49.8%) than PEO-Ti (42.4%) obviously due to the presence of calcium in its composition.

**Conclusion:**

The PEO-formed implant surfaces demonstrate high osseointegrative properties after any variants of treatment and show the potential for application in osteoporosis.

## Introduction

Dental implants (DI) have firmly occupied their place in the routine dental practice and provide predictable long-term results of prosthodontic treatment [1-5]. The analysis of the literature data indicates the presence of a large number of components promoting successful DI osseointegration, the predominant role belonging to the purity and microstructure of their surface [6, 7]. A high degree of roughness has been found to provide the mechanical stability of the implant at the time of its placement and in the long-term period of functioning [8–10]. The relief of the DI surface with the pores, having the depth and diameter of 1.5–4.0 μm, is recognized by the authors to be optimal for implant osseointegration [11, 12]. The DI with such a surface demonstrate the greatest resistance to unscrewing [13].

This study presents the results of application of plasma electrolytic oxidizing (PEO) technology to modify the surface of DI made from the medical VT1-0 titanium alloy; the structural and osseointegrative properties of the modified DI *in vivo* are also investigated.

**The aim of the study** is to evaluate the efficacy of the plasma electrolytic oxidation technology by comparing the results of two types of dental implants surface modification made of VT1-0 medical titanium alloy.

## Materials and Methods

### Preparation of samples and description of laboratory methods of investigation

 50 IRIS dental implants (Scientific Production Company LICOSTOM, Russia) 10-mm long and 4 mm in diameter were fabricated from VT1-0 alloy. Then, DI were divided into 2 equal groups and the implant surface of both groups underwent modification using the PEO technique in the sign-alternating electric field of a special form in two different electrolytes: group 1 — in the aqueous solution of alkaline electrolyte without any additional modifiers (POE-Ti); group 2 — in the aqueous solution of orthophosphoric acid-based electrolyte containing calcium carbonate (PEO-Ca).

Milled implants from VT1-0 titanium alloy with a smooth untreated surface served as control samples (n=10).

As soon as the process of coating was finished, all samples were washed in the bidistillate until no essential traces of foreign ions were found in the washing water, then packed in the zone of the laminar air flow (providing ISO 7 cleanliness) into the individual hermetic packages, numbered, and underwent gamma-sterilization.

For further investigations, DI with PEO in each of the two groups were distributed into subgroups using a random number generator. The surface of 10 implants (5+5) was examined by means of the scanning electron microscopy (SEM) (FlexSEM 1000 II; Hitachi, Japan) and energy-dispersive X-ray spectrometry (EDS) (Quantax 80; Bruker, Germany) with an automated acquisition of the element spectrum. The size and pore size distribution were also studied for these implants. This was done by analyzing the surface images using special software ICY 2.4.2.0 [14]. The pore surface concentration was defined as the ratio of their number to the sample area in the SEM image.

Other 10 (5+5) implants were tested for Vickers microhardness (HV) using NanoTest 600 Platform 3 nanoindentometer (Micro Materials LTD, Great Britain).

### In vivo investigation

 The remaining 30 implants with PEO-treated surfaces (15 PEO-Ti and 15 PEO-Ca) were installed into the sheep mandibular body by the extra-oral access following the standard surgical protocol. Investigations on animals were carried out at Stavropol State Medical University (Russia) and were approved by the local ethical committee (extract from protocol No.98 of May 20, 2021).

After 2, 4, and 8 weeks, the material was collected (3 DI from each sheep) and tested by means of microcomputed tomography. The Skyscan 1176 micro-CT scanner (Bruker microCT, Belgium) was used to examine the bone structure. The scanning parameters in the Skyscan 1176 program, v. 10.0.0.0, are as follows: radiograph voltage of 90 kV; radiograph current of 270 μA; 0.1-mm filter diameter; image pixel size of 17.74 μm; tomographer rotation of 360º; rotation pitch of 0.2; frame averaging of 4. The scanned objects were reconstructed in the Nrecon, v. 1.7.4.2 program (Bruker microCT, Belgium) using the following main parameters: smoothing of 2, circle reduction of 20, X-ray hardness of 41, contrast range of all images of 0.015–0.11. Spatial orientation (x, y, z) and selection of separate regions of the reconstructed material were performed using DataViewer program, v. 1.5.6.2 (Bruker microCT, Belgium).

## Results

### The results of electron microscopy of the implant surfaces and EDS spectroscopy

 Micrographs of DI with a smooth surface and after two types of treatment employing the PEO technology are presented.

**Figure 1.**
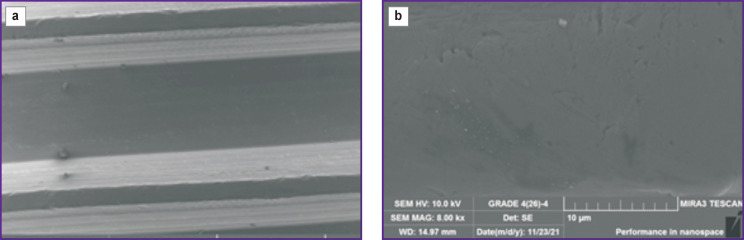
Microphotographs of the surface fragment of the dental implant from Grade IV alloy after milling without treatment of the initial surface: (a) ×127, (b) ×8000

The surface of the milled sample (Figure 1) represents a rather smooth surface with traces of mechanical processing. Chips and metal overflow are observed. The element composition of the untreated implant surface meets the standardized composition for the VT1-0 alloy.

A highly-developed surface with open micropores was formed on the PEO-modified samples irrespective of electrolyte composition, the surface area became 2.7 times larger relative to the milled implant.

**Figure 2.**
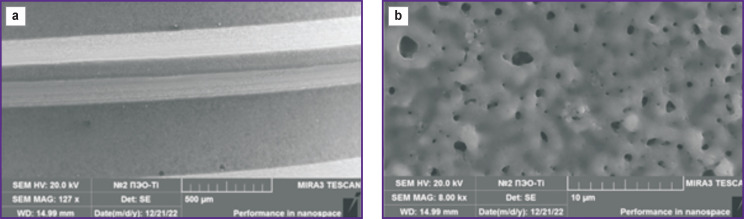
Microphotographs of the surface fragment of the dental implant from Grade IV alloy after treatment in PEO-Ti electrolyte: (a) ×127, (b) ×8000

The surface of the examined PEO-Ti samples after the treatment in the electrolyte represented a developed porous surface characterized by a large number of open pores (Figure 2). The pore sizes were distributed in a wide range from 200 nm to 3 μm. The pores had a cylindrical channel-like shape. At a large magnification (from ×16,000), crystallites about 50 nm in size could be observed on the sample surface. The main elements in the surface layer of the PEO-Ti samples were titanium (Ti) over 58.0 wt% and oxygen (O) about 40 wt%. Additionally, about 0.9 wt% of phosphorus (P) and less than 1.0 wt% of carbon (C), as well as insignificant amount of iron (Fe) less than 0.15 wt% and chlorine (Cl) less than 0.02 wt% were also detected.

**Figure 3.**
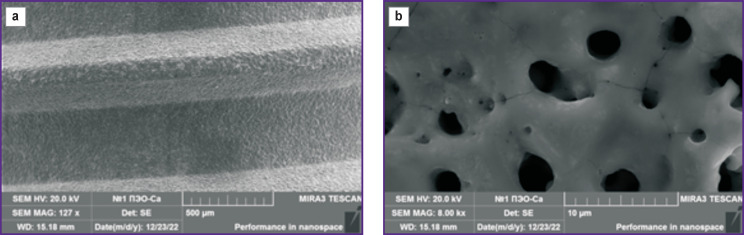
Microphotographs of the surface fragment of the dental implant from Grade IV alloy after PEO treatment in the electrolyte with Ca: (a) ×127, (b) ×8000

A developed porous surface of PEO-Ca is shown in Figure 3. The pores had a cylindrical channel-like structure. Their sizes varied in a wide range from about 80 nm to 7 μm. Toroid bumps were noted in some places where pores came to the surface. Cracks of ~55 nm thickness and, at a multiple magnification, a significant number of lamellar and needle-shaped particles could also be observed on the sample surfaces. The size of these particles was in the range from ~20 to ~150 nm. These particles are likely a solid residue formed after drying of the electrolyte solution.

The averaged data of the element composition of the examined implant surfaces obtained by EDS at a 1000-fold magnification are presented in Table 1.

**Table 1:** Element composition of the Grade IV sample at a 1000-fold magnification

Element	Surface type (wt%)			Surface type (wt%)		
	Without treatment	PEO-Ti	PEO-Ca	Without treatment	PEO-Ti	PEO-Ca
C	1.78	0.94	1.86	5.92	2.03	3.84
O	7.30	40.03	42.24	18.24	65.42	65.45
Ti	90.74	57.96	46.97	75.71	31.72	24.31
Fe	0.18	0.15	—	0.13	0.07	—
P	—	0.38	3.47	—	0.75	2.78
Ca	—	—	4.93	—	—	3.05
Na	—	—	0.53	—	—	0.57
Cl	—	0.02	—	—	0.02	—

### Pore distribution on the implant surfaces

 To analyze the pore size distribution, SEM images at a 3000-fold magnification and an 82-μm field of view were used. A monomodal distribution of pore sizes with about 0.23 μm maximum prevailed in the samples with the PEO-Ti surface. The diameter in this case varied within 0.2–3.0 μm.

The surface of the PEO-Ca treated implants possessed a wide range of size distribution from 0.08 to 7.0 μm. The pore distribution was of a bimodal character, in contrast to the PEO-Ti surface, with the main maximum of about 1.05 μm and the concomitant maximum near 2.45 μm.

The Vickers microhardness of the smooth VT1-0 titanium surface was 280 HV, the PEO-Ti modified surface — 400–800 HV, PEO-Ca — 360–480 HV.

### The results of in vivo implantation and osseointegration

**Figure 4.**
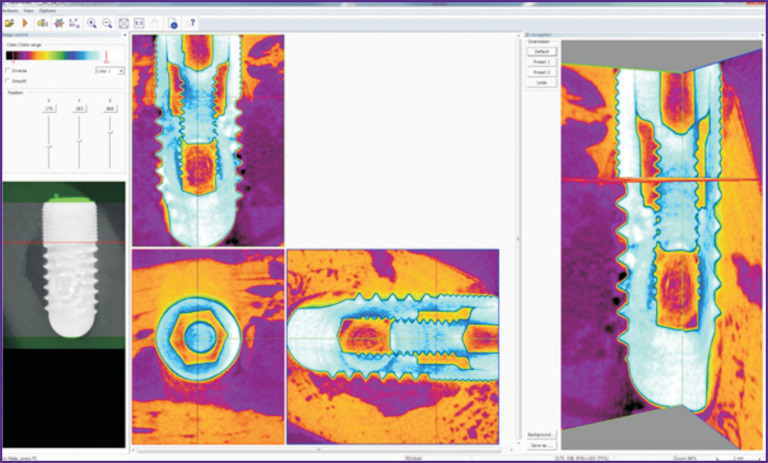
Visualization of the micro-CT data using the DataViewer program, multiplanar reformation *Orange color* — cortical bone in the region of the implant neck and apex, *violet color* — spongy bone in the area of the implant body

 After dental implantation, the wounds in all animals were healed by primary intention. There was no rejection and implant loss during the entire follow-up period. The micro-CT testing of the collected bone tissue with the implants has shown that all installed implants were embedded into the bone tissue, no exposed parts were found (Figure 4).

The volume occupied by the newly generated bone tissue of the region surrounding the implant (bone volume fraction: bone volume/total (tissue) volume — BV/TV, %) and bone-to-implant contact (BIC, %) are presented in Table 2.

**Table 2:** Maximum values of the examined indices

Investigated groups	A new generated volume of the surrounding the implant (≈900 μm from layer over the entire implant region), bone tissue the boundary BV/TV (%)			Bone-to-implant contact, BIC (%)		
	Day 15	Day 30	Day 60	Day 15	Day 30	Day 60
Milled implant VT1-0 (control)	31.8	14.4	20.0	42.7	20.8	25.0
PEO-Ti	46.9	20.0	32.9	46.2	30.9	42.4
PEO-Ca	52.6	18.3	32.6	52.4	40.8	49.8

## Discussion

Healing of the bone around the dental implants occurs according to the scheme of intramembranous osteogenesis with primary formation of the woven bone followed by remodeling and formation of the structured trabecular and compact bones. A newly generated bone is detected on the implant surface about 1 week after its installation. It is first observed in the region of the trabecular bone, later in the compact bone. Further remodeling of the bone tissue starts between the 6^th^ and 12^th^ weeks and goes on for a lifetime influenced by the functional load. Bone remodeling on the implant surface is a process of partial resorption and parallel generation of a new connection of the bone and implant. Surface modifications creating microroughness of implants accelerate the process of osseointegration of titanium implants, which has been demonstrated in multiple experiments on animals [9, 10, 15–17].

The main parameters of the DI surface influencing osseointegration are the character of roughness (topography) and a chemical composition. Although these parameters are often discussed independently, they are actually inseparable. Modern, well-documented implant systems with microrough surfaces demonstrate long-term survival rates [18]. The pore sizes within 1–10 μm are referred as microroughness. This range of microroughness maximizes the coupling between the mineralized bone and implant surface [15, 16, 19]. Hansson and Norton [8] supposed that an ideal implant surface must have semispherical pits with the diameter and depth from 1 to 5 μm and an ideal topographic relief capable of resisting the shear force at the bone–implant interface.

Plasma electrolytic oxidation, a promising domestic method of DI surface treatment developed by Beta-Tech Medicine LLC (Skolkovo, Russia) provides a high level of purity of these implants and their good osseointegrative properties. In the process of treatment, a hard oxide-ceramic coating with a porous structure adhered chemically to the metal base is formed on the titanium surface. The coating contains 41.66% of titanium (by atomic composition) from the implant material itself, which provides an excellent attachment to the surface. Additionally, the coating is doped with the atoms of calcium and phosphor for a fuller imitation of a natural bone tissue structure. In the process of treatment, the doping ions get into the coating from the oxidizing electrolyte and spread uniformly across the entire coating volume.

The results of our study show that the pore distribution over the surface of the examined implants coated according to the PEO technology in two types of electrolytes is in range of optimal size for obtaining osseointegration — 0.8–7.0 μm. However, the obtained implants differ in the distribution of the pores of various sizes over the surface. For example, the PEO-Ti surface has monomodal pore size distribution with the maximum near 0.23 μm. The PEO-Ca surface has a bimodal character, with the main maximum of 1.05 μm and a concomitant maximum of distribution near 2.45 μm. The sizes were found to vary widely from 0.8 to 7.0 μm. Similar results of surface morphology formed by PEO have been demonstrated by Kyrylenko et al. in their study [20].

The results of micro-CT investigation have demonstrated different values of a newly generated volume of the bone tissue surrounding the implant (BV/ TV, %) and the bone-implant contact (BIC, %). Thus, by day 14, BV/TV around the PEO-Ca implant was 52.6% against 46.9% for the PEO-Ti one. By day 30, this figure became 2 times less in both cases and increased again by the end of the second month reaching 32.9 and 32.6%, respectively. The value of BIC (%) by day 14 around the PEO-Ca was 46.2% against 52.4% for the PEO-Ti implant. By day 30, this value also decreased and grew again by the end of the second month approximating the values of day 15 — 42.4 and 49.8%, respectively. As has been reported by Jemat et al. [17], a rougher surface may facilitate faster attachment and proliferation of the osteogenetic cells. It should be noted that similar values for the milled (smooth) implant were 20% for BV/TV and 25% for BIC by the end of the second month.

Further histologic investigations may define the character of histological interaction of the bone tissue with the implant surface modified by the two types of PEO.

Several researchers have demonstrated that PEO coatings in combination with certain materials, including tricalcium phosphate, exhibit bioactive properties [21]. The porous structures of a nanometer scale are known to induce osseointegration [22-24], which may explain why the PEO coatings display good biological activity *in vivo* without any other modifications. Moreover, since a ceramic coating is “grown” on the implant surface, it is difficult to separate it from the titanium substrate; it provides excellent stability at the bone-implant interface and a reliable isolation of the main implant alloy [25].

Potential bioactive properties of the implant surfaces formed by the PEO method arouse great interest. Therefore, such implants are tested in deliberately complicated conditions — in osteoporosis. Polo et al. [26] managed to demonstrate that implants with PEO coatings in combination with calcium and phosphor ions installed to the rats with induced osteoporosis and low-quality bones facilitated bone formation and demonstrated high levels of bone maturation around the implants. Of special interest are the results of osseointegration investigation in the implants with PEO surface and those with the most widely spread type of surface modification by sandblasting and double acid etching (SLA). Momesso et al. [27] have shown in their work that computed microtomography, confocal microscopy, and medical history found similarity between the SLA and PEO surfaces with a tendency to PEO superioriority in animals with induced osteoporosis.

Thus, it has been shown that PEO surfaces demonstrate high osseointegrative properties whatever treatment is used and are perspective for application in osteoporosis.

## Conclusion

The laboratory and *in vivo* investigations have shown that PEO is a promising method of dental implant treatment. The obtained surfaces of various types (PEO with Ca and Ti) possess high purity and optimal microroughness for osseointegration: PEO-Ti — from 0.1 to 3.0 μm, PEO-Ca — from 0.08 to 7.0 μm. Both of the PEO treatment types have demonstrated similar osseointegrative potential, nevertheless, the PEO-Ca surface showed the best contact with the surrounding bone tissue (49.8%) relative to the PEO-Ti (42.4%) evidently owing to the presence of Ca in its composition.
